# Amalgam Solvents

**Published:** 1885-10

**Authors:** W. D. Miller


### AMALGAM SOLVENTS.

BY DR. W. D. MILLER.

The question of amalgam solvents raised by B. H. Teague (page 393 of this journal) is of sufficient interest to justify the presentation of certain observations which I have made in this direction. I am so situated that I have abundant opportunity to examine fillings of amalgam as well as of other materials, from almost every part of the world, and I have found one class of amalgam fillings, and only one, which invariably shows a disappearance of material from the surface, and sometimes so rapidly does the process go on that in five or six years the teeth require refilling. These fillings all contain copper, and as far as preserving teeth is concerned, they are vastly superior to any fillings of amalgam that I have ever seen. We constantly see fillings made of the finest modern amalgams, and inserted with great care, so imperfect that a good-sized excavator may be inserted between them and the walls of the cavity, but with the class of fillings above referred to this does not occur. The fillings are absolutely black or *reddish* black, as hard as glass and very brittle. On the grinding surfaces the material disappears very rapidly, so that in a few years often only a trace of amalgam will be found at the bottom of the cavity, but in all cases it clings to the walls of the cavity as though it had been melted and poured in. I never hesitate to fill up such cavities, over the amalgam, with gold. It makes an excellent foundation, and by its antiseptic power prevents the appearance of secondary decay. The idea that the two metals would give rise to disturbances in the pulp has no foundation, either in theory or practice. The cause of the washing away of these fillings is to me unknown; the fact, however, that it takes place more rapidly on the grinding than on the approximal surfaces would seem to indicate that the friction produced by mastication may possibly be one of the factors at work in bringing about this phenomenon. It is, however, certainly not the only cause, since fillings on the approximal and buccal surfaces undergo the same change, though in a somewhat slighter degree.

